# The Interplay of Students’ School Engagement, School Self-Concept and Motivational Relations during Adolescence

**DOI:** 10.3389/fpsyg.2017.02171

**Published:** 2017-12-13

**Authors:** Olga Bakadorova, Diana Raufelder

**Affiliations:** School Pedagogy, Institute of Education, Ernst-Moritz Arndt University Greifswald, Greifswald, Germany

**Keywords:** cross-lagged panel design, school engagement, school self-concept, teachers as positive motivators, peers as positive motivators

## Abstract

Existing literature evidences the association between adolescents’ school self-concept and engagement, both concepts being related to students’ perception of teachers and peers as motivators. However, few longitudinal studies explore the interplay of these factors. The present study aims to close this gap, applying latent cross-lagged panel design to two-wave data from German adolescent students [1088 8th grade students at T1 (*M*_age_ = 13.7, *SD* = 0.53; 53.9% girls) and 845 9th grade students at T2 (*M*_age_ = 14.86; *SD* = 0.57; 55% girls) from the initial sample]. Besides direct effects, three cross-lagged over-time paths were found to be significant: students’ perception of peers as positive motivators (PPMs) at the beginning of 8th grade (T1) positively predicts their behavioral school engagement at the end of 9th grade (T2), as well as emotional school engagement at the beginning of 8th grade positively predicts students’ perception of PPMs 1.5 years later. Furthermore, behavioral school engagement at T1 functions as a predictor of a student’s school self-concept at T2.

## Introduction

**FIGURE 1:**
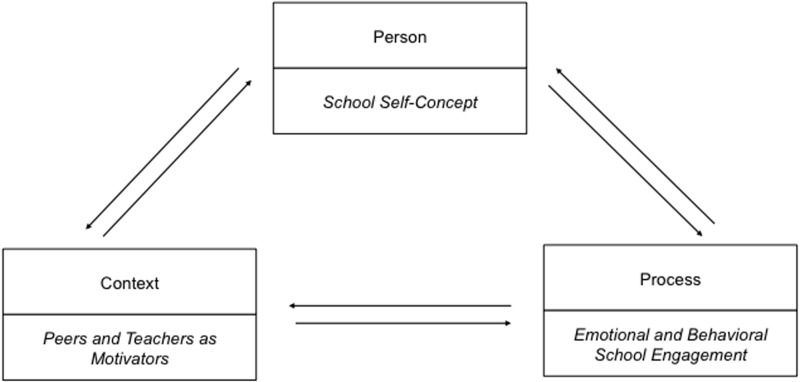
Design of the current study in accordance to the Developmental Ecological Model (Bronfenbrenner and Morris, 1998).

School engagement is an important factor in a student’s school career, as high engagement levels can enhance academic motivation and achievement (Connell et al., 1995; Fredricks et al., 2004; Appleton et al., 2008), whereas students’ disengagement may induce negative consequences as severe as school dropout (Finn, 1989; Fall and Roberts, 2012; Henry et al., 2012). The decline of emotional and behavioral school engagement in adolescence is a well-documented phenomenon in the modern world (Lam et al., 2016). At the same time, it is difficult to disentangle the factors that might support positive development of school engagement due to the fact that it is both influenced by environmental factors (Skinner and Belmont, 1993; Fredricks et al., 2004; Wang and Eccles, 2013) and vice versa (Skinner and Belmont, 1993; Van Ryzin, 2011), as well as by personal factors, particularly a student’s self-estimation of his or her abilities, intelligence and achievement related to school (Green et al., 2012), namely the school self-concept. This interplay between environmental and personal factors in the development of school engagement is in line with Bronfenbrenner’s Bioecological Theory (Bronfenbrenner, 1975, 1979; Bronfenbrenner and Morris, 1998), which states that the proximal environment (microsystem) (i.e., relationships with peers and teachers in school context) plays a key role in an individual’s development (see Veiga et al., 2012), as well as with the ideas of Lerner’s Developmental Contextualism (Lerner, 1986, 1991, 1992, 1998), in which the person-in-context is depicted as a function of dynamic processes embedded in reciprocal associations between a person and his or her contexts over time. Thereby, school engagement has been conceptualized “as part of a larger model of human motivation developed and elaborated over the last several decades (Deci and Ryan, 1985, 2000a; Connell and Wellborn, 1991; Skinner, 1991; Wellborn, 1991)" (Skinner et al., 2009, p. 495). In other words, the concept of school engagement is motivational in nature and therefore peers and teachers in their role as motivational supporters (Skinner and Belmont, 1993; Raufelder et al., 2015; see Skinner et al., 2009) are of special interest. Based on these theoretical assumptions, the developmental process of school engagement may be seen as an ongoing reciprocal interplay of personal factors (i.e., school self-concept) and the socio-motivational context (i.e., teachers and peers as positive motivators) (see **Figure 1**).

However, until today it is not clear whether these relations are in fact reciprocal within and over time through adolescence, or whether there is another clear (causal) order. This study aims to close this research gap and examine the within- and over-time associations of these variables from early to middle adolescence to gain a better understanding of the processes that accompany the trend of adolescents’ decline of engagement in school context. The findings might indicate potential starting points for prevention or intervention strategies to protect students from this downward trend.

### School Engagement

School engagement is defined as a complex and multidimensional construct (Appleton et al., 2008; Skinner et al., 2009), comprised of two to three components. The first component, (a) behavioral engagement, involves active participation in curricular and extracurricular school activities (Finn, 1993; Skinner and Belmont, 1993; Engels et al., 2016) as well as norm-conformant behavior or disobedience of school rules (Finn, 1993). The second component, (b) emotional engagement, denotes a student’s emotions and feelings toward teachers, peers and school in general (Skinner et al., 2009). This type of engagement especially supports students’ identification with their schools (Finn, 1989; Skinner and Belmont, 1993). Some of more recent models also include a third component (c) cognitive engagement, which might be defined as the “psychological investment” (Newmann et al., 1992; Fredricks et al., 2004) a student makes in his or her own learning process, thus possessing motivational properties (Fredricks et al., 2004). However, the current study follows the conceptualization and assessment of children’s behavioral and emotional engagement based on Skinner et al. (2009), who did not consider a cognitive component, because they used a motivational perspective on engagement (see Skinner et al., 2009). Furthermore, as this study aims to identify potential starting points for prevention and intervention strategies, the focus lies on the behavioral and the emotional components of school engagement, because research has shown that these components are considered especially important for practical interventions (Appleton et al., 2008; Christenson et al., 2008). Finally, the existing research taps at the differential effects of these components: while behavioral school engagement is predictive of school attendance and drop out-related outcomes (Connell et al., 1994), the same cannot be said about emotional school engagement (Rodríguez and Boutakidis, 2013).

The self-system model of motivational development (Connell, 1990) suggests that school engagement (a) has the behavioral and emotional components and (b) is, though not equal to motivation, yet motivational in nature (Connell and Wellborn, 1991; see Skinner et al., 2009; Reeve, 2012). This perspective underlines the role of teachers and peers in school context as important sources of students’ need satisfaction and motivation.

### School Engagement in the Socio-Motivational Context

As mentioned-above, the Bioecological Theory (Bronfenbrenner, 1975, 1979), Lerner’s Developmental Contextualism (Lerner, 1986, 1991, 1992, 1998) as well as later works (e.g., Pianta et al., 2012) promote the idea that school engagement is closely interrelated with the social context. At school, the social context is mainly constructed by teacher-student and student-student interaction. Both relationships with peers as well as with teachers are complex in nature: on a social level, peers and teachers constitute an important source of emotional support (Kindermann, 1993; Birch and Ladd, 1996, 1997, 1998; Juvonen and Wentzel, 1996; Azmitia et al., 2009; Jennings and Greenberg, 2009; Rubin et al., 2009; Eccles and Roeser, 2011), comfort and understanding (Ladd et al., 2009; Rubin et al., 2009). On a motivational level, teachers and peers have a major impact on students’ self-regulated motivation (Deci and Ryan, 1985) through the support of students’ need satisfaction as stated in the self-determination theory (Deci and Ryan, 1985): satisfying the three basic psychological needs (autonomy, competence, relatedness) is beneficial for a student’s motivation and high-quality engagement (Deci and Ryan, 2000a,b; Jang et al., 2009; Reeve, 2012). If a student perceives his or her peers and teachers as positive motivators (TPMs), it means that his or her motivation can be largely affected by his or her peers’ motivation, learning behavior or social support as well as through teachers’ motivation and perceived support (see Wentzel, 2009a,b; Raufelder et al., 2013a,b, 2016; Raufelder, 2014). Accordingly, several studies show that caring and motivating teachers may foster both behavioral (Battistich et al., 1997; Wang et al., 2013) and emotional (Furrer and Skinner, 2003) engagement in school-related activities. Moreover, some studies (e.g., Bennett et al., 1993) indicate that student’s engagement at school might have an effect on teachers’ beliefs in regard of a student.

Positive and motivating relations to peers are associated with an increase in both emotional and behavioral engagement at school (Wentzel, 2003). Longitudinal findings provide evidence that socio-motivational peer support during school transition predicts higher emotional and behavioral school engagement in secondary school (Li et al., 2011). Moreover, the level of socio-motivational support deriving from peers can be associated both with self-perception and engagement at school (Skinner et al., 2008; Li et al., 2011).

### School Self-Concept

According to the Bioecological Model of Bronfenbrenner (1975, 1979) and Lerner’s Developmental Contextualism (Lerner, 1986, 1991, 1992, 1998), it is important to account not only for the context, but also for the personal level, which in the school context may be manifested in the school self-concept. School self-concept is characterized by a student’s thoughts to own cognitive abilities in the school context (Schöne et al., 2012). In the course of adolescence, it is reported to be moderately stable (Gogol et al., 2016) and both associate with TPMs (Bakadorova and Raufelder, 2014) as well as school engagement (Green et al., 2012; Veiga et al., 2015). Some studies indicate that it is an important predictor of students’ school engagement (Locke, 2014; Raufelder et al., 2015), however, other sources state that it is hard to say whether it is a facilitator or an indicator of school engagement (Skinner and Pitzer, 2012).

### Aims and Hypothesis

In sum, the existing body of research suggests that (a) school self-concept, emotional and behavioral school engagement and teachers and peers as positive motivators might associate with one another and (b) that these associations might be bidirectional according to the ideas of the Bioecological Theory and Developmental Contextualism and (c) that they might exist not only within-time but also over-time (e.g., Skinner et al., 2008; Li et al., 2011; Veiga et al., 2015; Engels et al., 2016).

Accordingly, this study follows a cross-lagged panel research design to evaluate the interplay of school self-concept, socio-motivational relations with teachers and peers, and emotional and behavioral school engagement within- and over-time from the beginning of 8th grade to the end of 9th grade in secondary school context. Specifically, it was hypothesized that higher levels of school self-concept and a more positive perception of peers and TPMs would be concurrently and longitudinally related to higher levels of emotional and behavioral school engagement.

## Materials and Methods

### Participants and Procedure

The sample included 1088 8th grades students [8th grade, aged 12–15 (*M*_age_ = 13.7, *SD* = 0.53; 53.9% girls)] at Time 1 and remaining 845 students (*M*_age_ = 14.86; *SD* = 0.57; 55% girls) from the initial sample at Time 2 (1.5 years later at the end of 9th grade). This age group was chosen for two reasons: (1) As the school transition to secondary school occurs in Germany in 7th grade, students tend to struggle with the associated intra- and interindividual changes still at the beginning of 8th grade (Perkins, 1995; Parker, 2013). (2) Furthermore, students’ motivation tends to decline throughout the time of secondary school education (Kim et al., 2015) reaching its nadir in 9th grade (Eccles et al., 1998; Zusho and Pintrich, 2001; Watt, 2004). The participating students came from 23 randomly selected schools in the state of Brandenburg, Germany. Data on ethnicity was not collected due to the low proportion (2.6%) of ethnic minorities residing in this federal state. After the permission of state authorities was obtained, participants were selected based on the schools’, parents’ as well as their own consent. The voluntary and confidential nature of their involvement in this study was clearly communicated to all students involved. The data was collected by trained research instructors at the beginning of the German school year at T1 (beginning of 8th grade) on two consecutive days and at the end of 9th grade at T2. The participants received detailed instructions on how to complete the questionnaires and how to use Likert scales.

### Measures

*School Engagement* measures are based on the Engagement/ Disaffection Scales developed by Skinner et al. (2009). The BSE Scale (T1α = 0.75, T2α = 0.71) (e.g., “In class, I work as hard as I can”^1^) and the ESE Scale (T1α = 0.71, T2α = 0.65) (e.g., “I enjoy learning new things in class”) were comprised of six items each. Although the Cronbach alpha value for the ESE items at T2 was not as high as for the other subscales, parcels can be built according to Kopp and Lois (2012) statement that the critical value of Cronbach’s alpha is α > 0.50. Answers were rated on a four-point Likert Scale from (1) (strongly disagree) to (4) (strongly agree).

*School Self-Concept* (SSC) was addressed by a subscale of SESSKO scales developed by Schöne et al. (2002). The SSC subscale (T1α = 0.86; T2α = 0.87) consisted of five items measuring students’ perception of a rather general academic self-concept without any frame of reference (individual, social or criterial). Answers were rated on a five-point Likert scale, ranging from (1) “I am not gifted for school” or “In school tasks are difficult for me” to (5) “I am gifted for school” or “In school tasks are easy for me.”

The perception of *Teachers and Peers as Positive Motivators* was assessed using Relationship and Motivation (REMO) Scales (Raufelder et al., 2013a); (1) TPMs (T1α = 0.78; T2α = 0.79) subscale, featuring six items (e.g., “When a teacher takes his/her time to explain something to me, I will make more effort next time” or “When a teacher notices that I have tried my best, I will try to give my best again in the future”) and the (2) peers as positive motivators (PPMs) (T1α = 0.80; T2α = 0.82) subscale, including nine items (e.g., “My friends and I motivate each other to make an effort at school” or “When my friends learn, I am also motivated to learn more”). Responses for both subscales ranged from (1) “strongly disagree” to (4) “strongly agree” on an incremental four-point Likert-scale.

### Statistical Analyses

Initially, equality of item loadings and intercepts across time was tested, because measurement invariance is a precondition for cross-lagged panel design. The χ^2^-difference test was estimated using the Satorra–Bentler scaling correction factor (Satorra and Bentler, 2001) to test whether the sequentially imposed measurement invariance constraints lead to a significant decrease in the model fit. Afterward, the interplay between school engagement, socio-motivational relations and school self-concept were examined using a latent cross-lagged panel design (Finkel, 1995; Geiser, 2010) in Mplus with maximum likelihood estimation with robust standard errors (MLR) (Mplus 7.1; Muthén and Muthén, 1996–2012), which allows exploring the associations of variables in a within- and over-time perspective. The model included emotional school engagement, behavioral school engagement, school self-concept and socio-motivational relations with peers and teachers at T1 and T2, which were linked within time as well as over time, considering direct and cross-lagged paths.

There are several advantages for both psychometric characteristics as well as model estimation and fit characteristics to use parcel instead of single items: contrary to item-level data parcels have higher reliability, greater communality, higher ratio of common-to-unique factor variance, lower likelihood of distributional violations as well as more, tighter, and more-equal intervals, fewer parameter estimates, lower indicator-to-sample size ratio, lower likelihood of correlated residuals and dual factor loadings, as well as reduced sources of sampling error (Little et al., 2002; Nasser-Abu and Wisenbaker, 2006; Little et al., 2013). Accordingly, for each of the five latent variables, the items were randomly split into two parcels. Hence, the nine items of the PPM scale were transformed into two parcels consisting of four and five items each (PPM_P1, PPM_P2). The six items of the TPM, BSE, and ESE scale were transformed into two parcels with three items each (TPM_P1, TPM_P2; BSE_P1, BSE_P2, ESE_P1, ESE_P2); the five items of SSC scale were subdivided into two groups with three and two items each (SSC_P1, SSC_P2). Random parcel building is frequently used in psychological research (Nasser-Abu and Wisenbaker, 2006) to ensure that all measurement information is included in the structural equations.

We used the TYPE = COMPLEX function in Mplus to consider the classroom nesting of the data, because it supplies corrected standard errors and chi-square values regarding the nested structure of the data (1088 students in 71 school classes) (Asparouhov, 2005). Model fit was estimated by five primary fit indices, recommended by Hu and Bentler (1999): Chi-Square Test of Model Fit (χ^2^), Root Mean Square Error of Approximation (RMSEA), Comparative Fit Index (CFI), Tucker-Lewis Index (TLI) and Standardized Root Mean Square Residuals (SRMR). Respective CFI and TLI values above 0.95 and RMSEA and SRMR values up to 0.08 indicating an acceptable fit of the model. Due to the fact that missing data was completely at random (MCAR) as shown in Little’s (1988) MCAR test (χ^2^ = 117.35; *df* = 101; *p* > 0.05), missing data were handled using full-information maximum likelihood estimation (FIML).

## Results

### Descriptive Statistics and Bivariate Correlations

Bivariate correlations and descriptive statistics are reported in **Table 1**. Results from an unconditional latent change model (LCM) (Steyer et al., 1997; McArdle, 2009) that included all variables of interest demonstrated a significant decrease in BSE (Δ *M* = -0.10, *p* < 0.001, σΔ^2^ = 0.09, *p* < 0.001), ESE (Δ *M* = -0.05, *p* < 0.05; σΔ^2^ = 0.10, *p* < 0.001) and PPM (Δ *M* = -0.11, *p* < 0.001, σΔ^2^ = 0.20, *p* < 0.001). In turn, there was neither a significant mean decrease/increase in TPM (Δ *M* = -0.02, *p* > 0.05, σΔ^2^ = 0.19, *p* < 0.001) nor in SSC (Δ *M* = -0.03, *p* > 0.05, σΔ^2^ = 0.31, *p* < 0.001).

**Table 1:** Bivariate correlations and descriptive statistics of the constructs during both of the measurement points.

	BSE T2	ESE T1	ESE T2	SSC T1	SSC T2	PPM T1	PPM T2	TPM T1	TPM T2	Range	Mean	*SD*	Skewness (SE)	Kurtosis (SE)
BSE T1	0.48^∗∗^	0.66^∗∗^	0.40^∗∗^	0.38^∗∗^	0.29^∗∗^	0.11^∗∗^	0.04	0.21^∗∗^	0.12^∗∗^	1–4	2.79	0.49	-0.12 (0.08)	-0.07 (0.15)
BSE T2	–	0.36^∗∗^	0.61^∗∗^	0.21^∗∗^	0.31^∗∗^	0.14^∗∗^	0.07	0.11^∗∗^	0.14^∗∗^	1–4	2.74	0.45	-0.10 (0.09)	0.30 (0.17)
ESE T1		–	0.44^∗∗^	0.39^∗∗^	0.26^∗∗^	0.25^∗∗^	0.19^∗∗^	0.28^∗∗^	0.18^∗∗^	1–4	2.69	0.48	-0.37 (0.08)	0.74 (0.15)
ESE T2			–	0.18^∗∗^	0.34^∗∗^	0.16^∗∗^	0.20^∗∗^	0.10^∗∗^	0.21^∗∗^	1–4	2.71	0.44	-0.21 (0.09)	0.52 (0.17)
SSC T1				–	0.45^∗∗^	0.05	-0.01	0.11^∗∗^	0.04	1–5	3.47	0.62	-0.19 (0.08)	0.77 (0.15)
SSC T2					–	0.12^∗∗^	0.09^∗∗^	0.08^∗^	0.11^∗∗^	1–5	3.44	0.62	-0.32 (0.09)	1.02 (0.17)
PPM T1						–	0.42^∗∗^	0.41^∗∗^	0.24^∗∗^	1–4	2.55	0.51	-0.35 (0.07)	-0.06 (0.15)
PPM T2							–	0.20^∗∗^	0.39^∗∗^	1–4	2.46	0.41	-0.29 (0.08)	0.20 (0.17)
TPM T1								–	0.41^∗∗^	1–4	3.08	0.50	-0.38 (0.07)	0.34 (0.15)
TPM T2									–	1–4	3.06	0.49	-0.37 (0.08)	0.57 (0.17)


*All measures are standardized; ^∗^*p* < 0.05, ^∗∗^*p* < 0.01; BSE, behavioral school engagement; ESE, emotional school engagement; SSC, school self-concept; TPMs, teachers as positive motivators, PPMs, peers as positive motivators; T1, time 1 (beginning of 8th grade); T2, time 2 (end of 9th grade).*

Before conducting the cross-lagged panel design, a confirmatory factor analysis (CFA) was run. The CFA showed a good model fit [χ^2^(125) = 358.57, *p* < 0.001; *CFI* = 0.96, *TLI* = 0.95, *RMSEA* = 0.04 (0.04–0.05); *SRMR* = 0.03].

In order to test measurement invariance of all variables over time, we (1) specified an unconditional model (configural invariance) without equality constrains; (2) specified factor loadings as invariant over time (weak factorial invariance); and (3) set loadings and item intercepts invariant over time (strong factorial invariance) (see **Table 2**). Strong measurement invariance over time has been found, supporting the assumption that the constructs remained stable over time and therefore, flavoring the use of cross-lagged panel design (Kenny, 2005; Geiser, 2010).

**Table 2:** Measurement invariance.

Measurement invariance								

Model	χ^2^	df	*p*	RMSEA	90%CI	CFI	TLI	SRMR
Model 1	358.58	125	<0.001	0.04	0.04–0.05	0.96	0.95	0.03
Model 2	363.86	130	<0.001	0.04	0.04–0.05	0.96	0.95	0.04
Model 3	370.21	135	<0.001	0.04	0.04–0.05	0.96	0.95	0.04

**Step**	**Model**			**Δχ^2^**	***p***	**Δdf**		

Step 1	Configural invariance			–	–	–		
Step 2	Weak invariance			3.39	0.64	5		
Step 3	Strong invariance			5.43	0.37	5		


*Model 1 = no constraints but configural invariance; Model 2 = loadings invariant across time; Model 3 = loadings and intercepts invariant across time.*

### Cross-Lagged Panel Design

**FIGURE 2:**
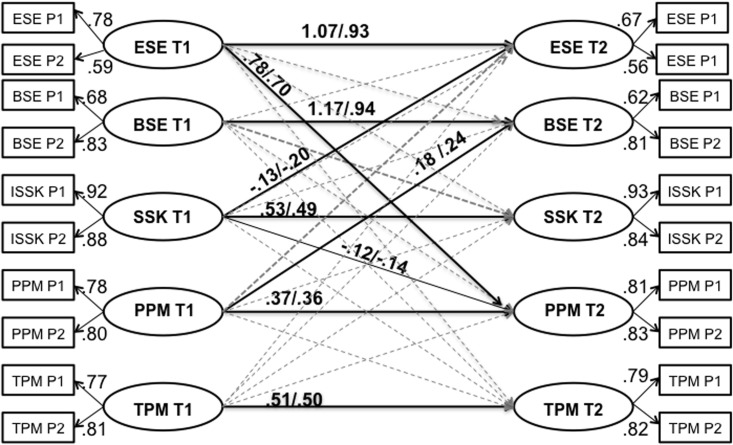
Cross-lagged panel design: ESE, emotional school engagement; BSE, behavioral school engagement; SSC, school self-concept; PPM, peers as positive motivators; TPM, teachers as positive motivators; P1, parcel 1; P2, parcel 2. T1, time 1; T2, time 2 (1.5 years later). Significant effects shown as unstandardized coefficients (B) in first position and standardized coefficients (β) in second position; bold pathways are significant at *p* < 0.05; dotted pathways are not significant. Covariances between all variables within each wave (T1 and T2) are not shown in the figure for reasons of clarity but reported in the text.

The final cross-lagged panel design model (see **Figure 2**) showed a good fit χ^2^(123) = 308.842, *p* < 0.001, *CFI* = 0.97, *TLI* = 0.96, *RMSEA* = 0.04 (0.03–0.05), *SRMR* = 0.03.

#### Within-Time Associations

The within-time associations between TPM and PPM are positively significant (T1: *r* = 0.10/0.52; *p* < 0.001; T2: *r* = 0.06/0.45; *p* < 0.001) as well as the within-time associations between BSE and ESE (T1: *r* = 0.14/0.90; *p* < 0.001; T2: *r* = 0.08/0.89; *p* < 0.001) Furthermore, TPM is positively associated with BSE (T1: *r* = 0.05/0.30; *p* < 0.001; T2: *r* = 0.02/0.21; *p* < 0.05) and ESE (T1: *r* = 0.07/0.41; *p* < 0.001; T2: *r* = 0.03/0.35; *p* < 0.01). In turn, the association between PPM and BSE (T1: *r* = 0.03/0.18; *p* < 0.001) and between PPM and ESE (T1: *r* = 0.07/0.38; *p* < 0.001) was significant solely at T1. In addition, the association between SSC and BSE (T1: *r* = 0.10/0.52; *p* < 0.001; T2: *r* = 0.04/0.31; *p* < 0.001) as well as the association between SSC and ESE (T1: *r* = 0.12/0.53; *p* < 0.001; T2: *r* = 0.07/0.56; *p* < 0.001) was found to be positively significant. In contrast, there was no significant relation between SSC and PPM neither at T1 nor at T2. In turn, a positive significant association between SSC and TPM was found (T1: *r* = 0.03/0.14; *p* < 0.01; T2: *r* = 0.02/0.11; *p* < 0.05).

#### Over-Time Associations: Direct Effects

The model evidenced positive direct effects of each variable from T1 to T2 supporting the stability of the constructs: BSE (*B* = 1.17, β = 0.94, *SE* = 0.41, *p <* 0.01), ESE (*B* = 1.07, β = 0.93, *SE* = 0.46, *p* < 0.05), TPM (*B* = 0.51, β = 0.50, *SE* = 0.07, *p* < 0.001), PPM (*B* = 0.37, β = 0.36, *SE* = 0.11, *p* < 0.001), SSC (*B* = 0.53, β = 0.49, *SE* = 0.06, *p* < 0.001).

#### Over-Time Associations: Cross-Lagged Effects

Students’ perception of PPM in early adolescence predicted BSE in middle adolescence (*B* = 0.18, β = 0.24, *SE* = 0.09, *p* < 0.05). Furthermore, ESE at T1 positively predicted PPM at T2 (*B* = 0.78, β = 0.70, *SE* = 0.35, *p* < 0.05). In turn, SSC at T1 negatively predicted both ESE (*B* = -0.13, β = -0.20, *SE* = 0.08, *p* < 0.01) and PPM at T2 (*B* = -0.12, β = -0.43, *SE* = 0.06, *p* < 0.05).

## Discussion

Following the ideas of Bronfenbrenner’s (1975, 1979) Bioecological Model and Lerner’s (1986) Developmental Contextualism, which state an ongoing bidirectional relationships between person, context and process, this study set out to discover the within- and over-time interplay of students’ school self-concept (person), the perception of teachers and peers as positive motivators (context) and both behavioral and emotional school engagement (process) from the beginning of 8th grade to the end of 9th grade in secondary schools. The major aim was to identify potential starting points for prevention and intervention strategies against the decrease in school engagement in school context during adolescence.

In line with the hypothesis and existing empirical research (Furrer and Skinner, 2003; Green et al., 2012; Wang et al., 2013), the results of the within-time associations showed that both adolescents’ behavioral and emotional school engagement were positively associated with school self-concept as well as with the perception of TPMs at both waves. This finding contradicts existing research by suggesting that the association of school self-concept and engagement might decline in middle adolescence (Veiga et al., 2015) and hence indicating that the role of school self-concept in adolescence might be underestimated. However, we found a negative over-time association between school self-concept and emotional school engagement. In other words, although there is a positive association between school self-concept and emotional engagement at both T1 and T2, the long-term effect of school self-concept on emotional engagement is negative. This means, that students with a high school self-concept at the beginning of 8th grade show less emotional participation in class at the end of 9th grade. One reason for this negative over-time association might be that students with a high school self-concept get bored more quickly and therefore get more emotionally frustrated with class over time, which enhances the findings from Veiga et al. (2015), who showed that middle adolescents with high self-concept lost their prior high levels of cognitive and agentic engagement. However, this negative over-time relationship might also be a result of a suppression effect (Paulhus et al., 2004) and vertical multicollinearity problems, which can occur while using correlated subscales from the same construct (i.e., school engagement) (Kock and Lynn, 2012), as based on the correlation analysis, this association is positive.

Besides, the significant mean change of school engagement supports the existing research, which showed – similar to the motivational decline at the beginning of early adolescence – a downward tendency in students’ school engagement as well (Lam et al., 2016). The significant mean decrease in students’ perception of TPMs enhances the research that showed that students tend to perceive their teachers as more distant and cold during secondary school compared to elementary school (Eccles et al., 1993; Harter, 1996), as well as the research that showed that students, studying in the grades six to eight perceive their teachers as more evaluative and controlling (Harter, 1996).

As a practical implication, teachers should be aware of their role as positive motivators and its impact as there is a positive association of TPMs with both school self-concept and school engagement at both waves. In other words, teachers, who are motivated themselves and provide support to their students, are not only an important source of students’ motivation but rather an essential source in the prevention of a downward tendency in adolescents’ school engagement. This finding supports Skinner et al.’s (2009) conceptualization of school engagement as a motivational one, highlighting the positive associations between a teacher’s motivational support and school engagement (Skinner and Belmont, 1993). However, it should be noted that – against our hypothesis – there is no significant over-time path from TPMs to any other variable in this study, which means that a teacher’s impact on school-engagement and school self-concept seems to be more effective in the short-term perspective. This finding underlines the differentiated effects of different levels in the relationship between teachers and students, as research that examined the social (not motivational) level of teacher-student relations prioritized their role in the enhancement of students’ engagement (e.g., Pianta et al., 2012; Engels et al., 2016), behavioral school engagement in particular (Engels et al., 2016). Accordingly, measures that are more focused on the interpersonal level, on which teachers disclose their more general approval or disapproval of the student as a person (Birch and Ladd, 1996), that could affect a student’s sense of identity (Birch and Ladd, 1998; Alerby and Hertting, 2007; Jennings and Greenberg, 2009) could lead to different results. Future studies are necessary to consider these both levels. Another possible explanation for this phenomenon might be the period of 1.5 years between T1 and T2 including potential teacher change from beginning of 8th grade to the end of 9th grade. Furthermore, the REMO scale used in this study to measure students’ perception of TPMs follows a more general approach, as students were asked to think of their teachers in general. Future studies are warranted, which use measures of students’ perception of the motivational support of one specific teacher over time (i.e., TEMO scale developed by Raufelder and Hoferichter, 2015).

In contrast to our hypothesis, there was a positive significant within-time association between students’ perception of their behavioral school engagement and PPMs at T1, but not at T2. However, the over-time associations of the cross-lagged panel design show that especially the perception of PPMs at T1 plays an important role for later behavioral engagement support, implying a potential causal relationship, such as peers in their role of positive motivators influencing behavioral school engagement, but not vice versa. These findings might indicate that the role of PPMs is particularly important in early adolescence with short-term and long-term effects on (at least behavioral) school engagement, whereas this role generally decreases in importance during middle adolescence. This finding is in accordance with research that showed that peers as secondary socialization instance are particularly important in early adolescence, when students turn away from family (Fend, 1998; Brown and Theobald, 1999; Cook et al., 2007) and have to deal with school transition accompanied by colder and more distant teacher-student relationships (Eccles et al., 1993; Harter, 1996). However, it is possible that in middle adolescence the role of the peer group as a source of motivation gradually decreases: while directly after the school transition students need their peers’ support and acceptance to get better integrated into a new setting (Juvonen and Cadigan, 2002), after some time they might find other sources of motivation. Indeed, the existing research taps that adolescents’ relations with peers are subject to continuous change (e.g., Cairns and Cairns, 1994; Ryan, 2001). The over-time association between PPMs and behavioral school engagement, in turn, expands the existing findings about the long-term influence of peers on behavioral engagement in adolescence (e.g., Demo, 1992) and the importance of establishing positive motivational relationships with peers directly after the transition to secondary school as this could (expanding the findings of Wentzel, 1998; Perdue et al., 2009; Veiga et al., 2014) not only yield more active behavioral involvement at that period of time, but also be a facilitator of school-related activities in the future. In turn, emotional school engagement at T1 positively predicted students’ perception of PPMs at T2, suggesting that positive emotions toward school, the class and important others in the school context may result in more positive perceptions of PPMs in the future. This can be explained by the nature of emotional school engagement, which, in accordance with Fredricks et al. (2004), involves not only affective reactions to school and school-related activities, but also emotional reactions to teachers and students. Therefore, it is not surprising that positive emotions related to certain classmates result in positive motivational relations with them over the course from early to middle adolescence.

In addition, there was neither a significant within-time association between PPMs and school self-concept at T1 nor at T2. This means that adolescents’ school self-concept is less sensitive to motivational support from peers compared to motivational support from teachers (see above). This may be due to the fact that teachers in their institutional role possess more opportunities to promote a student’s academic self-concept: TPMs may not only satisfy students’ affiliative needs, but also support the needs of competence and autonomy by clear set goals, well-articulated expectations, meaningful instructions and empowerment (Roeser et al., 1998; Pianta et al., 2003; Radel et al., 2010). In turn, and against our hypothesis, school self-concept negatively predicts students’ perception of PPMs over time. In other words, the higher early adolescents’ school self-concept at the beginning of 8th grade is, the less he or she perceives or needs their peers as a source of motivation at the end of 9th grade. This finding is in line with results from a qualitative study, which showed that adolescent students with a high school self-concept feel more motivated through comparison to others in terms of competition with the aim to “win the race” (Bakadorova and Raufelder, 2015).

## Conclusion

Overall, our two-wave cross-lagged panel design study showed that the within-time interplay between school self-concept, behavioral and emotional school engagement as well as students’ perception of peers and TPMs tend to be stronger in early adolescence at the beginning of 8th grade than in middle adolescence at the end of 9th grade. This might be explained by a growing need for autonomy from adults during middle adolescence (Eccles and Midgley, 1989; Midgley, 1993) and increased skills in self-regulated learning and motivation processes with less support from peers and teachers, as well as possible changes in peer relations over the span of 1.5 years (Cairns and Cairns, 1994; Ryan, 2001). Our study expands the existing findings of long-term relations among the above-mentioned factors and suggests that the explored interplay is not reciprocal in nature, but rather (causally) ordered, which should be tested in longitudinal studies with more than two waves in more detail.

In particular, the role of PPMs seems to loose impact from early to middle adolescence: while PPMs were positively associated with both behavioral and emotional school engagement at T1 and positively associated over time with behavioral school engagement, there was no significant association of PPMs and behavioral school engagement at T2. However, there was a positive over-time association between emotional school engagement at 8th grade and students’ perceptions of PPMs at the end of 9th grade, not only supporting the existing findings that these two factors positively associate (Juvonen and Wentzel, 1996), but also underlining the possible positive effects of emotional school engagement on peer relationships. In addition, there are within-time associations between TPMs and both emotional and behavioral school engagement at both waves, although no over-time association could be identified. Nevertheless, this implies that motivated and supportive teachers are an essential preventive factor in the downward tendency of school engagement in adolescence.

In sum, our findings underline the relevance of Bronfenbrenner’s Bioecological Theory and Lerner’s Developmental Contextualism in the explored interplay in a short-term perspective, such as peers and teachers play differentiated roles for different aspects (i.e., school self-concept, emotional, and behavioral school engagement) at different times during adolescence and vice versa. In other words, the development of adolescent students’ school engagement is embedded in complex dynamics between a student’s sense of their own abilities in school and their motivational relationships with peers and teachers within-time, although no relation between the variables is bidirectional over-time. Therefore, fostering both students’ school self-concepts as well as their motivational relations with peers and teachers might benefit their emotional and behavioral school engagement and vice versa in the course of adolescence, whereas the beneficial effect is greater the earlier it starts.

### Strengths, Limitations, and Further Directions

The current research evidences several theoretical and methodological limitations. First, although students’ subjective perceptions were at the heart of the study, self-report is often subject to criticism. However, some authors (Spector, 2006; Chan, 2009) claim that the problems associated with self-report data equally apply to non-self-report data. Second, the cognitive engagement mentioned by numerous authors was not included in the current research, as we followed the conceptualization and assessment by Skinner et al. (2009). However, future replication studies might transfer the study design on other conceptualization of school engagement including the cognitive component. Third, there are limitations in the psychometric quality of the variable ESE. Although this measure proved to have good psychometric qualities when used in other studies with different populations, it showed restricted psychometric qualities in the present sample at T2. However, due to its substantial contribution to the present model, we decided not to remove the variables. In addition, the research relies solely on students’ data; teachers’ evaluations, data from school psychologists and/or social workers and parents could assist a triangulation of perspectives, and are thus advisable variables to be taken into account for future investigations. Future research should be planned to draw conclusions about the international generalizability of the research results. Finally, while one may criticize that this study focuses on general school self-concept rather than on subject-related school self-concepts (i.e., verbal vs. math school self-concept), school engagement as well as students’ perception of peers and teachers as motivators are based on general school context rather than specific subjects as well, which justifies our choice.

At the same time, the current study evidences a number of strengths. Firstly, it focuses on absolute school self-concept, allowing practical interventions through school psychologists, as impact on only one aspect of school self-concept within school environment (verbal or mathematic) might induce negative changes in the counterpart (e.g., Niepel et al., 2014). Secondly, it applies the ideas of the Bioecological Theory of Bronfenbrenner (1975, 1979) as well as Lerner’s (1991) Developmental Contextualism to the secondary school setting (in contrast to e.g., Skinner and Belmont, 1993; Perdue et al., 2009) and considers both the motivational roles of teachers and peers, school self-concept and different aspects of school engagement (both behavioral and emotional) over the course of 1.5 years. The cross-lagged longitudinal research design we used for this aim presents a clear statistical strength of the study (Little et al., 2002), as it allows discovering patterns of variables within and over time (Burkholder and Harlow, 2003). Finally, the large number of participating students and schools combined with repeated data collection allows us to generalize our findings for the state of Brandenburg, Germany.

## Ethics Statement

This study was carried out in accordance with the recommendations of the guidelines of the Ministry of Education, Youth and Sports of Brandenburg (http://bravors.brandenburg.de/verordnungen/wissuv_1998) with written informed consent from all subjects. All subjects gave written informed consent in accordance with the Declaration of Helsinki. The protocol was approved by the Ministry of Education, Youth and Sports of Brandenburg.

## Author Contributions

All authors agree to be accountable for the content of the work. OB did the statistical analyses and wrote the main part of the paper. DR assisted with the statistical analyses and reviewed the paper, contributed decisively to the discussion part and added comments on the manuscript throughout the process of manuscript development.

## Conflict of Interest Statement

The authors declare that the research was conducted in the absence of any commercial or financial relationships that could be construed as a potential conflict of interest.

## Footnotes

1 All original German items have been translated into English for the current paper in order to provide illustration for an international reader.
